# Supplementary material for: Heterodimerization of Munc13 C2A domain with RIM regulates synaptic vesicle docking and priming
Source: Nat Commun. 2017 May 10;8:15293. doi: 10.1038/ncomms15293 (PMC5436228; doi:10.1038/ncomms15293)
Supplement: Supplementary Information — Supplementary Figures and Supplementary Table. [file ncomms15293-s1.pdf]

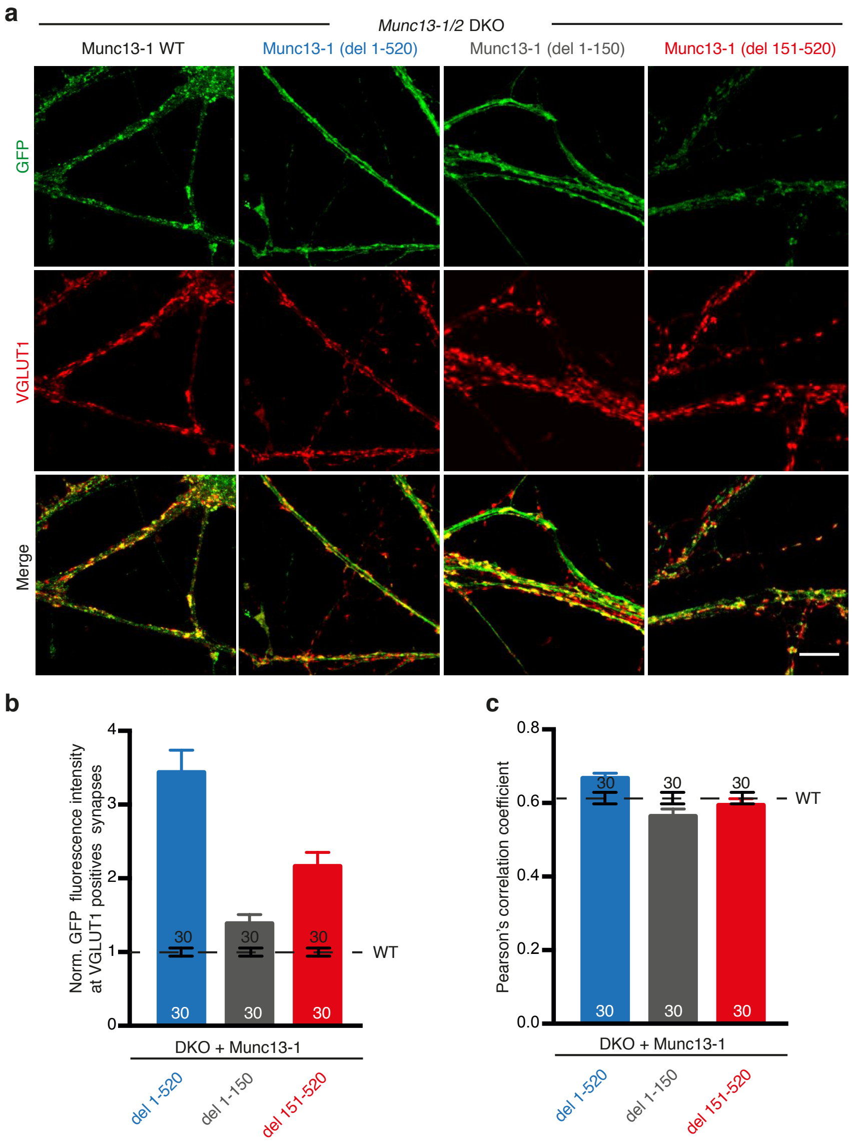

Supplementary Figure 1: Quantification of Munc13-1 WT and deleted mutants at the presynaptic terminals. (**a**) Maximum projection images of *Munc13-1/2* DKO hippocampal neurons rescued with Munc13-1 WT or C_2A mutants: Munc13-1 (del 1-520), Munc13-1 (del 1-150) and Munc13-1 (del 151-520) showing the double labeling for GFP and VGLUT1. Each row of images shows the labeling of GFP (green upper panel), VGLUT1 (red middle panel) and merge (bottom panel). Scale bar, 10 μm. (**b**) Plot of GFP fluorescent intensity levels of Munc13-1 mutants at VGLUT1 positives compartments normalized to WT Munc13-1 intensity levels (dotted line). GFP mean intensity values were analyzed in 50 positives synapses per cell, in 10 different cells per group in 3 independent cultures, that correspond to 1500 synapses per group. (**c**) Plot of the Pearson's correlation coefficients between GFP and VGLUT1 signal for all C_2A mutants normalized to WT Munc13-1.

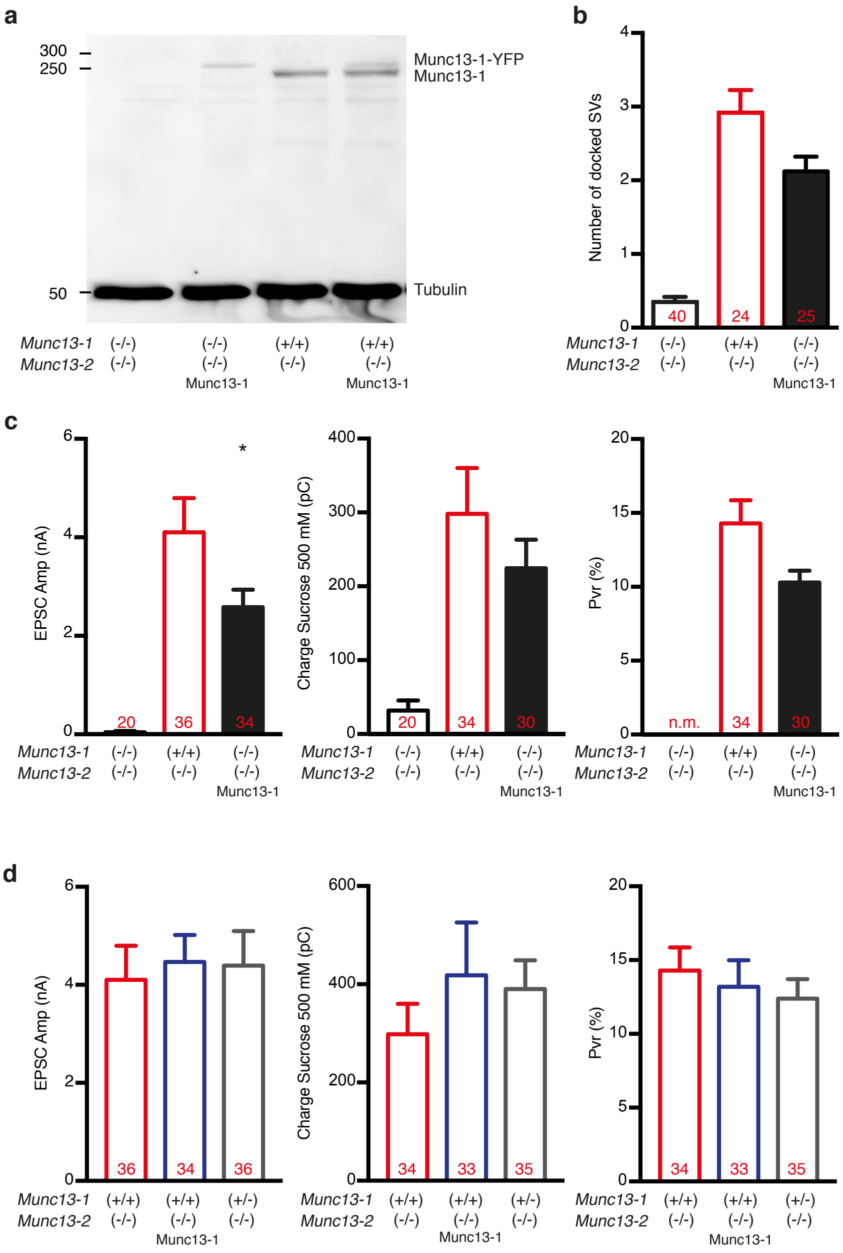

Supplementary Figure 2: Synaptic transmission in Munc13-1/2 DKO neurons rescued with Munc13-1 WT and in neurons with alterations of the Munc13-1 levels. (**a**) Immunoblot showing Munc13-1 expression levels in hippocampal neurons from *Munc13-1/2* DKO, *Munc13-1/2* DKO rescued with Munc13-1, *Munc13-1*$^{+/+}$/*Munc13-2*$^{/-}$ and *Munc13-1*$^{+/+}$/*Munc13-2*$^{/-}$ with overexpression of Munc13-1. The antibody used for detection was panMunc13 antibody. Molecular weights (kDa) are indicated on the left side. Note the difference in position of both endogenous Munc13-1 and Munc13-1-YFP. (**b**) Plot of number of docked SVs from *Munc13-1/2* DKO, *Munc13-1*$^{+/+}$/*Munc13-2*$^{/-}$ and *Munc13-1/2* DKO rescued with Munc13-1 neurons. (**c**) Summary plots of average EPSC amplitudes, RRP charge and p_{vr} in *Munc13-1/2* DKO (black bar graph)., *Munc13-*

1$^{+/+}$/*Munc13-2*$^{-/-}$ (red bar graph) and *Munc13-1/2* DKO rescued with Munc13-1 neurons (black filled bar graph). (**d**) Summary plots of average EPSC amplitudes, RRP charge and p_{vr} in *Munc13-1*$^{+/+}$/*Munc13-2*$^{-/-}$ (red bar graph), *Munc13-1*$^{+/+}$/*Munc13-2*$^{-/-}$ with overexpression of Munc13-1(blue bar graph), *Munc13-1*$^{+/-}$ /*Munc13-2*$^{-/-}$ (grey bar graph). Numbers in bar graphs are *n* values for each group. n.m. non measurable. Significances and *p* values were determined by One-way ANOVA with Kruskal-Wallis test followed by Dunn's post-test. Values indicate mean ± SEM; **, $p < 0.01$.

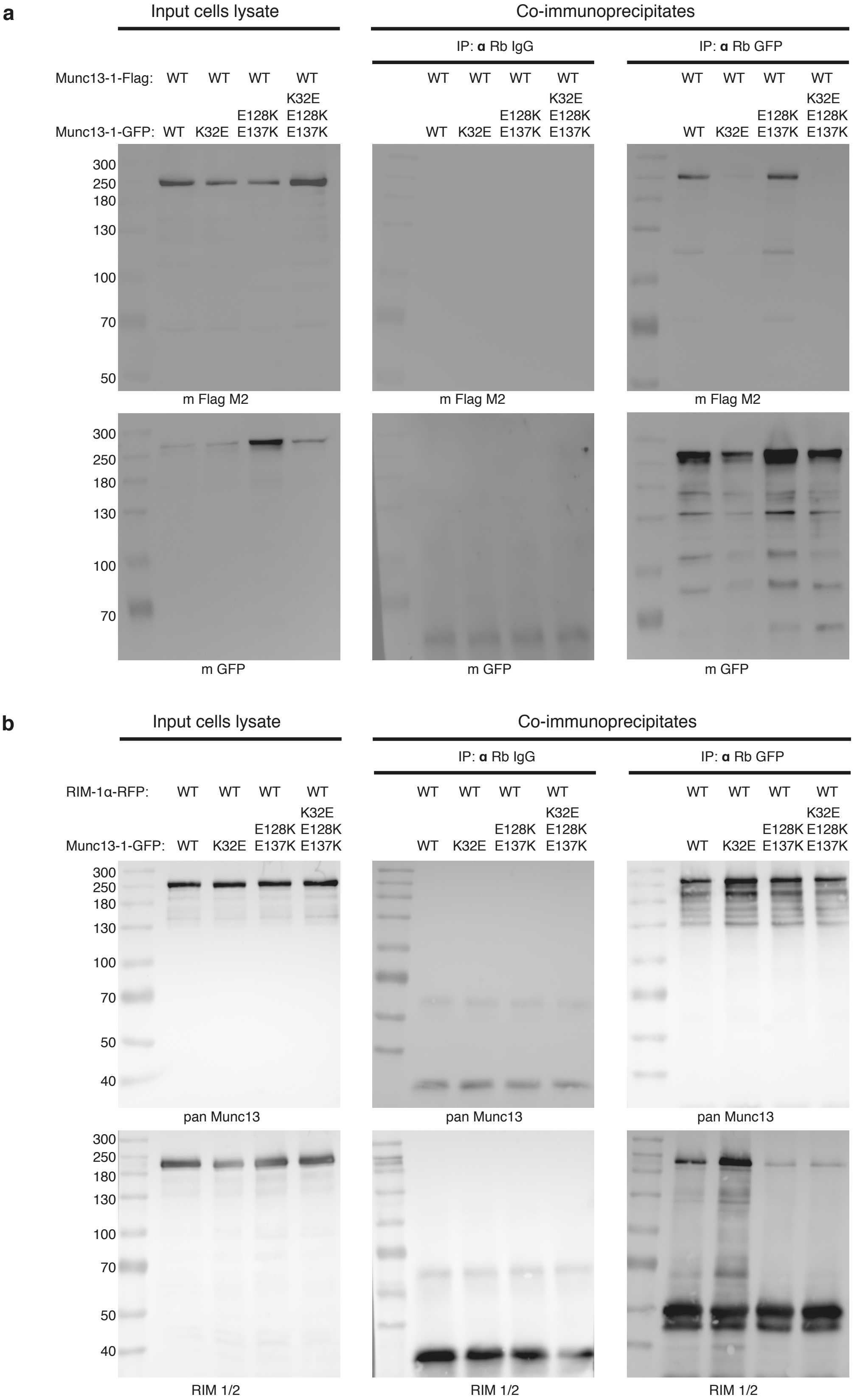
a
Input cells lysate
Co-immunoprecipitates
IP: α Rb IgG
IP: α Rb GFP
Munc13-1-Flag: WT WT WT WT
K32E
E128K E128K
Munc13-1-GFP: WT K32E E137K E137K
300
250
180
130
100
70
50
m Flag M2
m GFP
b
Input cells lysate
Co-immunoprecipitates
IP: α Rb IgG
IP: α Rb GFP
RIM-1α-RFP: WT WT WT WT
K32E
E128K E128K
Munc13-1-GFP: WT K32E E137K E137K
300
250
180
130
100
70
50
40
pan Munc13
RIM 1/2

Supplementary Figure 3: Homodimeric and heterodimeric interactions of Munc13 C_2A domain. (**a**) CoIP of Munc13-1-GFP in HEK293 cells transiently double-transfected with Munc13-1-Flag and Munc13-1 WT or mutants tagged with GFP. Left plots corresponded to detection of the input lysates, middle panels are detection of the proteins of interest after the co-immunoprecipitation with the control IgG antibody and right plots corresponded to detection of the proteins after the co-immunoprecipitation with the Rb GFP antibody. (**b**) CoIP of RIM-1α in HEK293 cells transiently double-transfected with RIM-1α-RFP and WT or mutants Munc13-1 GFP tagged. Left plots corresponded to detection of the input lysates, middle panels are detection of the proteins of interest after the co-immunoprecipitation with the control IgG antibody and right plots corresponded to detection of the proteins after the co-immunoprecipitation with the Rb GFP antibody. These results are representative of three independent experiments. Molecular weights (kDa) are indicated on the left side and antibodies use for detection on the bottom side of each blot. Antibody used for the immunoprecipitation is showing on the top.

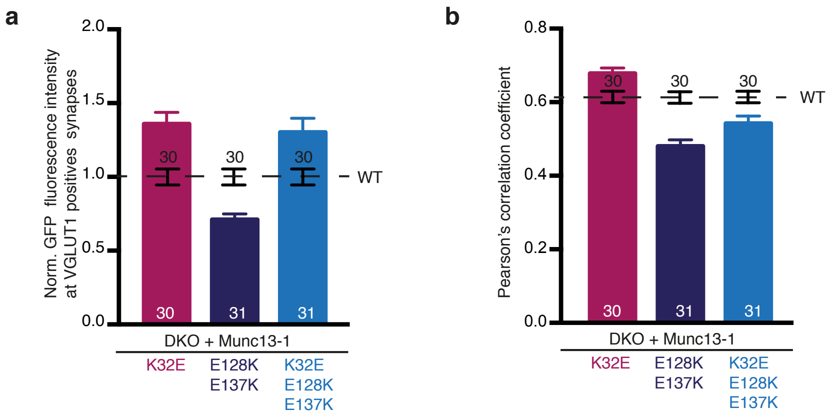

Supplementary Figure 4: Quantification of presynaptic expression levels of Munc13-1 WT and Munc13-1 C_2A domain point mutants. (**a**) Plot of GFP fluorescent intensity levels of Munc13-1 C_2A point mutants within VGLUT1 positives compartments normalized to Munc13-1 WT expression (dotted line). GFP mean intensity values were analyzed in 50 positives synapses per cell, in a total of 30 different cells per group from three independent cultures that correspond to 1500 synapses per group. (**b**) Plot of the Pearson's correlation coefficients between GFP and VGLUT1 signal for all Munc13-1 C_2A point mutants. Data were collected from at least three independent cultures. Numbers in bar graphs are *n* values for each group. Error bars represent SEM.

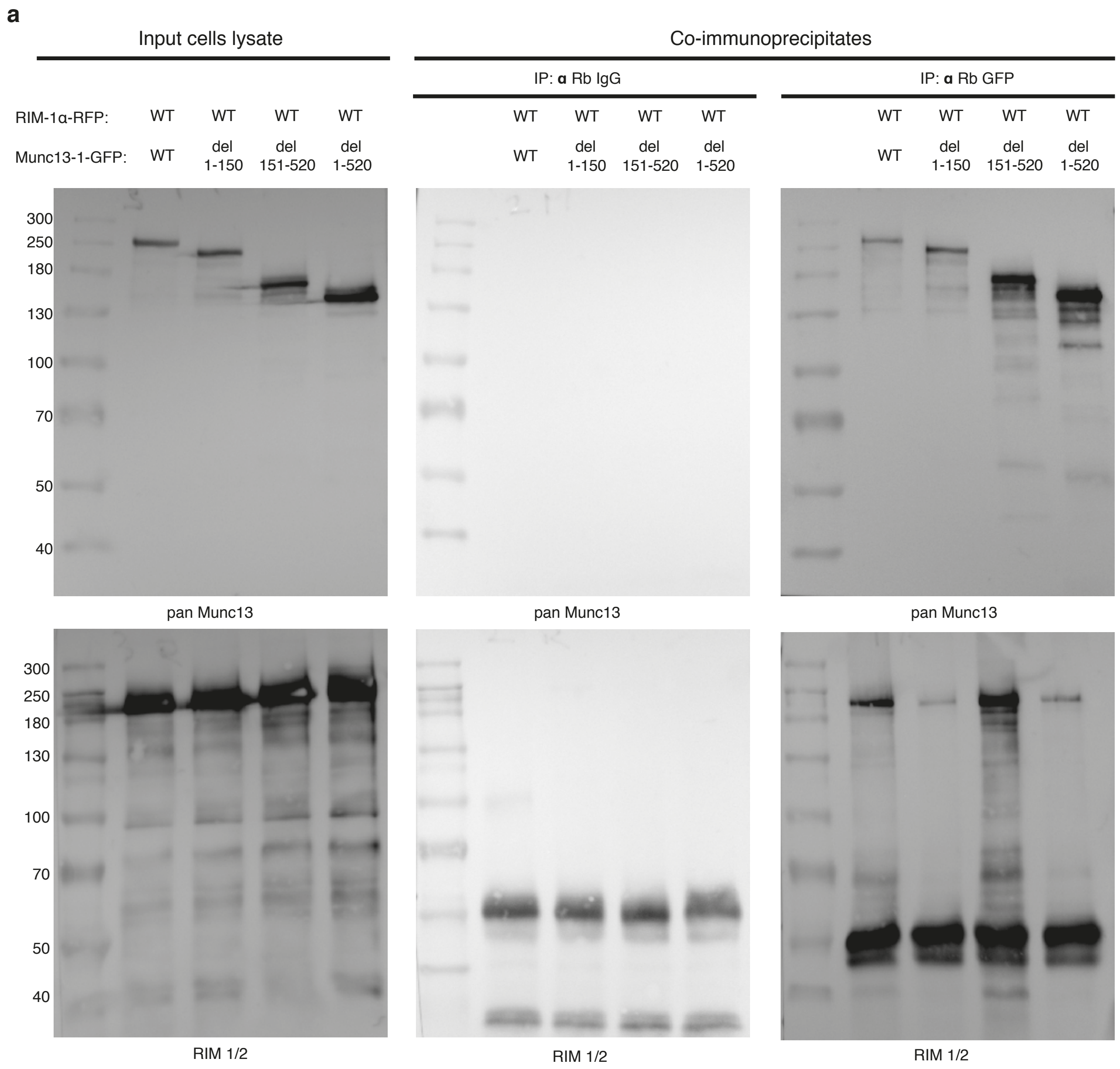

Supplementary Figure 5: Elimination of C_2A domain of Munc13 does not completely disrupt the interaction of RIM-1α. (a) CoIP of RIM-1α in HEK293 cells transiently double-transfected with RIM-1α-RFP and Munc13-1 WT or mutants GFP tagged. Left plots corresponded to detection of the input lysates, middle panels are detection of the proteins of interest after the co-immunoprecipitation with the control IgG antibody and right plots corresponded to detection of the proteins after the co-immunoprecipitation with the Rb GFP antibody. These results are representative of three independent experiments. Molecular weights (kDa) are indicated on the left side and antibodies use for detection on the bottom side of each blot. Antibody used for the immunoprecipitation is shows at the top.

Supplementary Table 1: Ultrastructural analyses of synaptic vesicles in synapses rescued with Munc13-1 N-terminal deletion and C_2A domain point mutants.

Sample	n	SV diameter	# of SV within 100 nm of AZ	AZ length
DKO	126	36.2 ± 0.3	10.68 ± 0.4	376.5 ± 9.3
+ Munc13-1 WT	99	36.2 ± 0.3	12.72 ± 0.5	362.1 ± 9.0
+ Munc13-1 del 1-150	111	35.0 ± 0.3	12.62 ± 0.4	365.0 ± 11.9
+ Munc13-1 del 1-520	111	36.2 ± 0.2	13.20 ± 0.5	400.6 ± 10.9
+ Munc13-1 del 151-520	69	37.9 ± 0.4	11.77 ± 0.6	398.7 ± 13.0
+ Munc13-1 K32E	86	36.2 ± 0.2	12.14 ± 0.5	396.0 ± 11.6
+ Munc13-1 E128K,E137K	115	36.9 ± 0.3	11.39 ± 0.4	399.6 ± 14.8
+ Munc13-1 K32E,E128K,E137K	115	36.84 ± 0.3	10.67 ± 0.4	398.5± 15.6

DKO: Munc13-1/2 double knockout; del: deletion; SV: Synaptic vesicle; AZ: active zone; n: number of synaptic profiles.
